# First Reported Use of the AMDS Hybrid Prosthesis for Secondary Type A Aortic Dissection After Prior TEVAR

**DOI:** 10.3390/jcdd13030141

**Published:** 2026-03-18

**Authors:** Gjoko Boshkoski, Dorgam Natour, Atanas Jankulovski, Thomas Felderhoff, Aron. F. Popov

**Affiliations:** Department of Cardiovascular Surgery, Asklepios Clinic Harburg, 21075 Hamburg, Germany; d.natour@asklepios.com (D.N.); a.jankulovski@asklepios.com (A.J.); t.felderhoff@asklepios.com (T.F.); a.popov@asklepios.com (A.F.P.)

**Keywords:** aorta diseases, type A aortic dissection, type B aortic dissection, TEVAR, AMDS stent, ascending aorta replacement, aortic arch repair, hybrid aortic surgery

## Abstract

Type A aortic dissection represents one of the most life-threatening cardiovascular emergencies, with management strategies evolving toward hybrid and endovascular approaches, particularly in high-risk patients. The Ascyrus Medical Dissection Stent (AMDS) is an emerging adjunctive technology designed to promote true lumen expansion and facilitate favorable aortic remodeling during open repair of acute Type A dissection. We present the first reported case of AMDS deployment in secondary Type A dissection following prior thoracic endovascular aortic repair (TEVAR). An 83-year-old female with extensive aortic history—including TEVAR in 2012 for intramural hematoma with chimney stenting to the left subclavian artery and carotid–subclavian bypass in 2013—developed acute Type A dissection extending into the existing stent graft in 2024. Emergency surgical intervention included ascending aortic replacement, aortic arch repair with AMDS implantation, aortic valve resuspension, and left atrial appendage resection under cardiopulmonary bypass and hypothermic circulatory arrest. Postoperative imaging confirmed appropriate AMDS positioning, false lumen exclusion, and preservation of prior endograft integrity. The patient tolerated the procedure well and was discharged in stable condition with favorable early follow-up outcomes. This case demonstrates the potential role of hybrid surgical strategies and adjunctive endovascular devices in managing complex, multi-stage aortic disease.

## 1. Introduction

Acute aortic syndromes (AAS)—comprising aortic dissection, intramural hematoma, and penetrating aortic ulcer—are among the most lethal emergencies in cardiovascular medicine, with high early mortality if not promptly recognized and treated [1]. Of these, acute Type A aortic dissection (ATAAD) poses the most immediate threat, frequently leading to catastrophic outcomes such as rupture, cardiac tamponade, or organ malperfusion without emergent surgical intervention [2].

Recent advancements in imaging modalities, particularly computed tomography angiography (CTA), have significantly enhanced the speed and accuracy of AAS diagnosis, enabling timely triage and operative planning [3]. In parallel, management strategies have evolved to incorporate both open and endovascular techniques, often combined in hybrid approaches tailored to the complexity of the underlying pathology [4]. Thoracic endovascular aortic repair (TEVAR) has become the standard of care for complicated Type B aortic dissections, especially in patients with high surgical risk profiles [5].

The Ascyrus Medical Dissection Stent (AMDS) is a novel hybrid prosthesis developed to augment open surgical repair of ATAAD. By supporting distal anastomosis, promoting true lumen expansion, and encouraging favorable remodeling of the aortic arch and proximal descending aorta, AMDS offers a less invasive alternative to total arch replacement or frozen elephant trunk (FET) procedures [6,7].

Early clinical outcomes with AMDS have been encouraging. In the PERSEVERE trial—a prospective, multicenter study conducted across 26 U.S. institutions—93 patients underwent open surgical repair of ATAAD with AMDS implantation. Clinical malperfusion was present in 82% of patients and radiographic malperfusion in 18%. At 30 days, the composite rate of major adverse events was 27%, notably lower than the 58% observed in historical controls. Importantly, there were no distal anastomotic new entry tears, and the technical success rate reached 99%. Early postoperative imaging demonstrated stabilization of aortic diameter, true lumen expansion, and regression of the false lumen in the treated segment. These findings underscore the feasibility and potential benefit of AMDS, particularly in patients presenting with malperfusion syndromes [8].

However, the application of AMDS in patients with a prior thoracic endograft presents unique anatomical and technical challenges—including altered aortic compliance, disrupted laminar flow, and limited landing zones—that complicate distal anastomosis in reoperative settings and require careful consideration of prior endograft geometry. To our knowledge, no prior cases have been reported describing the use of AMDS in the setting of a secondary Type A dissection following previous TEVAR for a Type B aortic pathology. This report presents the first such case, highlighting the technical feasibility and early success of this hybrid strategy in the management of progressive, multi-stage aortic disease.

## 2. Case Presentation

An 83-year-old female with a history of complex aortic disease presented to the emergency department with acute-onset left-sided facial paresis, accompanied by severe anterior chest pain, hypotension, and signs of impending hemodynamic compromise. Her past medical history was notable for hypertension and extensive thoracoabdominal aortic pathology.

In 2012, she underwent thoracic endovascular aortic repair (TEVAR) for an extensive intramural hematoma involving both the thoracic and abdominal segments of the aorta. To preserve perfusion to the left upper extremity, a chimney stent was simultaneously placed in the left subclavian artery. One year later, in 2013, she required a left carotid–subclavian bypass to address a dissection of the subclavian artery, a known complication of complex thoracic aortic interventions [9,10].

**Figure 1:**
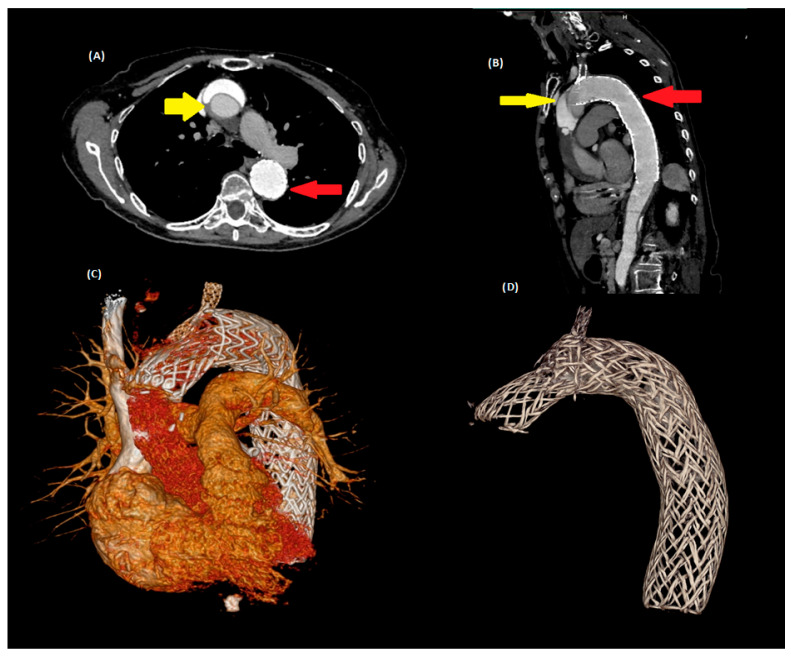
Contrast-enhanced computed tomography and three-dimensional reconstructions. (**A**) Axial view showing the intimal flap of the ascending aorta (yellow arrow) and the thoracic endovascular aortic repair (TEVAR) stent graft in the descending aorta (red arrow). (**B**) Sagittal view demonstrating extension of the Type A dissection into the previously implanted TEVAR stent graft. (**C**) Three-dimensional reconstruction illustrating intraoperative positioning of the (AMDS) relative to the TEVAR stent graft. (**D**) Postoperative three-dimensional reconstruction confirming appropriate deployment of the AMDS.

Twelve years after the index TEVAR, the patient re-presented with clinical features concerning for acute aortic syndrome. Emergency contrast-enhanced computed tomography angiography (CTA) demonstrated a Stanford Type A aortic dissection originating from the ascending aorta, with extension into the previously placed thoracic stent graft (Figure 1A,B). The dissection flap propagated distally to the upper abdominal aorta, with evidence of true lumen compression and suspicion for early visceral malperfusion. The primary intimal tear was identified at the level of the mid-ascending aorta, proximal to the brachiocephalic artery (BCA). The BCA and left common carotid artery (LCCA) were free of dissection on cross-sectional imaging, with preserved true lumen perfusion. The left subclavian artery (LSA) was excluded from the acute dissection anatomy by the pre-existing carotid–subclavian bypass, which was confirmed patent. The dissection did not extend into the carotid arteries. Emergency cerebral computed tomography (CT) was performed given the patient’s presentation with acute-onset left-sided facial paresis; no acute ischemic infarction was identified, and the neurological symptom was attributed to transient hemodynamic compromise rather than structural cerebrovascular occlusion. The prior TEVAR stent graft had been implanted in the proximal descending aorta with a landing zone in zone 3, with a proximal oversizing of approximately 15%; no significant migration or collapse of the endograft was observed.

Given the patient’s advanced age, prior aortic intervention, and the complexity of the dissection anatomy, a multidisciplinary aortic team recommended emergent hybrid surgical repair. Under general anaesthesia, a median sternotomy was performed. Cardiopulmonary bypass was established, and moderate hypothermic circulatory arrest with antegrade cerebral perfusion was initiated. The ascending aorta was replaced using a prosthetic Dacron graft. At the distal anastomosis, an AMDS was deployed to stabilize the dissected aortic arch, facilitate re-expansion of the true lumen, and optimize distal aortic remodeling—particularly at the junction with the prior endograft [7,11] (Figure 1C,D). Arterial cannulation was performed via the right axillary artery to facilitate antegrade perfusion and enable antegrade cerebral perfusion; venous drainage was established via the right atrium. Bilateral antegrade cerebral perfusion was delivered through direct cannulation of the BCA and LCCA at flows of 13–16 mL/kg/min, maintaining a target cerebral perfusion pressure of 70 mmHg. Total cardiopulmonary bypass time was 139 min; circulatory arrest was performed at 22 °C (deep hypothermia) for a total of 24 min. Preoperative risk stratification using the EuroSCORE II yielded a predicted mortality of 79.3%. An AMDS 55/55 device (Atrivion, Inc., Atlanta, GA, USA)was selected based on the diameter of the native distal aortic arch and the proximal edge of the existing TEVAR stent graft. The distal anastomosis was fashioned at the proximal arch, in direct continuity with the proximal edge of the pre-existing TEVAR stent graft, allowing the self-expanding AMDS to telescope into and overlap with the endograft by approximately 116–131 mm, ensuring an adequate sealing zone. No additional reinforcement sutures or surgical felt pledgets were required at the anastomotic line. Postoperative CTA confirmed exclusion of the false lumen at the visceral segment level, with patent true lumen perfusion to all major visceral branches, including the celiac axis, superior mesenteric artery, and bilateral renal arteries.

Concomitantly, resuspension of the aortic valve commissures was performed to correct leaflet prolapse and restore valve competence. The left atrial appendage was resected prophylactically due to permanent atrial fibrillation (AFib) in order to reduce the long-term risk of thromboembolic events in this high-risk patient.

The patient tolerated the procedure without intraoperative complications. Her postoperative course was uneventful, with preserved neurologic function, hemodynamic stability, and no signs of end-organ dysfunction. Follow-up CTA confirmed correct positioning of the AMDS, durable exclusion of the false lumen, and stable integration with the previously implanted thoracic stent graft. The patient was discharged in stable condition on postoperative day 12 and remained clinically well at early follow-up (6 weeks postoperatively).

## 3. Discussion

This case highlights a rare and technically complex clinical scenario: the development of a secondary Stanford Type A aortic dissection (ATAAD) in a patient more than a decade after thoracic endovascular aortic repair (TEVAR) for an extensive intramural hematoma. The successful use of the AMDS in this setting represents a novel hybrid approach to aortic arch repair in the context of prior stent grafting—an application not previously described in the literature.

The AMDS is a hybrid open surgical prosthesis designed to simplify distal anastomosis during open repair of ATAAD, while also promoting true lumen expansion and favorable aortic remodeling. It enables a conservative hemiarch replacement while stabilizing the dissected arch, without requiring total arch replacement or the frozen elephant trunk (FET) technique [7,11].

The FET procedure has long been employed in complex aortic arch and descending thoracic aortic pathologies, offering a single-stage solution that combines open and endovascular repair. However, FET is associated with prolonged operative and circulatory arrest times, increased risk of spinal cord ischemia, and technical challenges related to graft delivery and sealing [6,12]. In contrast, AMDS is deployed directly through the open arch and can significantly reduce circulatory arrest time, which is particularly valuable in elderly or high-risk patients such as the one presented here [2]. While early studies have confirmed the safety and efficacy of AMDS in primary ATAAD, its use in reoperative or hybrid contexts, especially following prior TEVAR, has not been previously documented.

Retrograde dissection following TEVAR is an uncommon but serious complication, with an incidence estimated at 1–3% [13]. Proposed mechanisms include excessive proximal stent oversizing, trauma at the landing zone, and progression of underlying aortic disease [14]. In this case, the dissection extended retrogradely from the ascending aorta into the prior thoracic stent graft, presenting several diagnostic and operative challenges.

High-resolution computed tomography angiography (CTA) played a central role in defining the dissection morphology and guiding surgical strategy. The rigid endograft limited standard distal anastomosis options and increased the complexity of transitioning from native aorta to prosthetic graft. The AMDS, with its self-expanding stent design and open arch compatibility, provided an ideal solution for bridging the dissected arch to the prior TEVAR segment while facilitating true lumen expansion and remodeling.

Emerging evidence continues to support the versatility of AMDS in complex aortic surgery. A recent multicenter study by Piks et al. evaluated AMDS in a “one-fits-all” strategy for ATAAD and reported favorable early results in technical success, aortic remodeling, and complication rates [15]. Although their cohort did not include patients with prior TEVAR, their findings reinforce the adaptability and safety profile of AMDS in diverse anatomical contexts—further substantiating its application in complex or reoperative settings, as demonstrated in our case. An important patient selection consideration when applying AMDS is the status of the supra-aortic vessels (SAVs). Approximately 30% of ATAAD cases demonstrate dissection extending into the BCA, LCCA, or LSA [16]. In such cases, AMDS—which targets proximal aortic stabilization without dedicated SAV reconstruction—may be insufficient to exclude the false lumen at the arch vessel level, potentially sustaining the risk of thromboembolic stroke or malperfusion. For patients with significant SAV dissection, total arch replacement with the FET technique may offer more reliable vessel-by-vessel exclusion and cerebral protection. In our patient, the BCA and LCCA were free of dissection, and the LSA was anatomically addressed by the pre-existing carotid–subclavian bypass, eliminating the risk of untreated SAV false lumen perfusion. This favorable anatomy was integral to the decision to use AMDS and should be considered a prerequisite for its safe application. Furthermore, the one-year PERSEVERE results (Szeto et al., 2025) reported four cases of potential distal stent graft-induced new entry tears (SINE); in the post-TEVAR context, the pre-existing endograft may reduce this risk by mechanically stabilizing the distal landing zone [16].

This case illustrates several potentially important clinical observations, though conclusions must be interpreted cautiously given the single-case design. First, it suggests that AMDS may be feasible in reoperative settings involving existing thoracic endografts, expanding the range of scenarios in which this device might be considered. Second, it highlights the potential value of hybrid approaches in complex aortic disease, particularly when conventional techniques such as FET carry prohibitive risk. Finally, it reinforces the importance of lifelong imaging surveillance following TEVAR, as secondary proximal complications—including ATAAD—can emerge years after the initial intervention [5]. Multicenter data and prospective registries will be needed before broader practice recommendations can be made.

In comparison with FET, AMDS offers a simplified and potentially safer method for distal arch stabilization in select patients. The technique reduces operative time and eliminates the need for total arch replacement, which is particularly advantageous in frail or elderly patients. Although further studies are needed to assess long-term outcomes, this case illustrates how novel adjuncts like the AMDS can be safely integrated into complex aortic reconstructions, even in anatomically altered or previously treated aortas. In this patient, the decision not to proceed with FET was driven by several specific factors: her advanced age (83 years), severely elevated operative risk, and the presence of a pre-existing endograft in the descending aorta, which would have necessitated complex device-in-device interactions and potentially prolonged circulatory arrest times. FET requires careful sizing relative to pre-existing endografts and carries inherent risks of retrograde Type A dissection, spinal cord injury, and incomplete sealing in the presence of prior stent grafts [6,12]. The AMDS, in contrast, permitted a controlled, expedient distal anastomosis without the need for total arch replacement, and its flexible self-expanding design allowed it to conform to the pre-existing endograft geometry. Importantly, not every patient with ATAAD is a candidate for AMDS. Contraindications or relative limitations include dissection extending into the SAVs without additional reconstruction, significant arch pathology requiring vessel reimplantation, and anatomical configurations incompatible with the available device sizes. Careful patient selection—including preoperative CTA assessment of arch anatomy, SAV status, and endograft configuration—is essential. Regarding the risk of stent-induced new entry (SINE), this is a recognized complication following TEVAR and has also been observed in the context of AMDS deployment. In the PERSEVERE one-year follow-up, four cases of potential SINE were identified [16]. In our case, the pre-existing thoracic endograft occupied the distal landing zone of the AMDS, potentially reducing the risk of SINE by mechanically stabilizing the aortic wall at this level and minimizing unsupported dissected segments. Nonetheless, this risk cannot be excluded and warrants close surveillance imaging.

## 4. Learning Points

- This is the first documented case of AMDS implantation following prior TEVAR, used in the management of a secondary Type A aortic dissection.
- AMDS offers a feasible and less invasive alternative to total arch replacement or FET in high-risk or reoperative patients.
- Retrograde dissection after TEVAR, although rare, remains a serious late complication that requires careful imaging surveillance and tailored surgical planning.

## 5. Conclusions

This case underscores the feasibility and clinical utility of the AMDS as an adjunct in complex aortic surgery, specifically in the context of reoperative arch repair following prior TEVAR. To our knowledge, this is the first reported instance of AMDS implantation for secondary Stanford Type A aortic dissection occurring after TEVAR for Type B pathology. The successful application of this hybrid approach highlights the evolving role of adjunctive endovascular technologies in extending the therapeutic options for patients with multistage, anatomically complex aortic disease. Serial CTA surveillance is planned at 3, 6, and 12 months postoperatively to monitor aortic remodeling, carotid–subclavian bypass patency, and overall endograft integrity, in accordance with our institutional protocol for complex aortic reconstructions. Reporting followed the CARE guidelines (Supplementary Materials).

## 6. Patient Perspective

The patient expressed gratitude for the prompt diagnosis and comprehensive care she received. She was initially unaware of the severity of her condition but appreciated the clarity of communication and surgical explanation provided by the clinical team. Following recovery, she reported improved well-being and expressed confidence in the outcome and the decision-making process that led to her treatment.

## Data Availability

The original contributions presented in this study are included in the article. Further inquiries can be directed to the corresponding author.

## Supplementary Materials

The following supporting information can be downloaded at: https://www.mdpi.com/article/10.3390/jcdd13030141/s1, The CARE checklist. Reference [17] is cited in the supplementary materials.
