# Supplementary material for: First Reported Use of the AMDS Hybrid Prosthesis for Secondary Type A Aortic Dissection After Prior TEVAR
Source: J Cardiovasc Dev Dis. 2026 Mar 18;13(3):141. doi: 10.3390/jcdd13030141 (PMC13027261; doi:10.3390/jcdd13030141)
Supplement: Supplementary file 1 [file jcdd-13-00141-s001.zip › jcdd-4147121-supplementary.pdf]

CARE Case Report Checklist

Manuscript ID: jcdd-4147121 | Journal: JCDD | Date: February 26, 2026

Title: "First Reported Use of the AMDS Hybrid Prosthesis for Secondary Type A Aortic Dissection After Prior TEVAR"

Authors: Boshkoski G, Natour D, Jankulovski A, Felderhoff T, Popov AF

This checklist was completed in accordance with the CARE guidelines (Gagnier et al., J Med Case Rep, 2013; BMJ, 2013). All 13 checklist items are addressed below with the corresponding manuscript location.

#	Section	CARE Checklist Item	Present in Manuscript	Page
**Title / Abstract**				
1	Title	The words case report should appear in the title.	✓ Title states "First Reported Use..." — manuscript type identified as 'Case report' in the section header above the title.	1
2	Abstract	Key elements of the case: the main symptoms of the patient, the main diagnoses, the main interventions, and the main outcomes.	✓ Abstract includes: presentation (acute Type A dissection, prior TEVAR), intervention (ascending replacement + AMDS), and outcomes (discharge POD 12, stable follow-up).	1
**Introduction**				
3	Introduction	One or two paragraphs summarising why this case is unique and the related medical literature.	✓ Introduction reviews AMDS background, PERSEVERE trial data, and explains why AMDS in the post-TEVAR setting has not previously been described.	1–2
**Patient Information**				
4a	Patient information	De-identified patient demographic information (age, sex, ethnicity, occupation).	✓ 83-year-old female. No ethnicity or occupation stated (not clinically relevant to the case).	2
4b	Patient information	Chief complaints of the patient (primary symptoms for which the patient sought medical care).	✓ Acute-onset left-sided facial paresis, severe anterior chest pain, hypotension, impending haemodynamic compromise.	2
4c	Patient information	Medical, family, and psychosocial history including relevant genetic information.	✓ Hypertension; prior TEVAR (2012, intramural hematoma); chimney stent left subclavian (2012); left carotid–subclavian bypass (2013); permanent atrial fibrillation. No family/genetic history relevant to the case.	2
4d	Patient information	Relevant past interventions with outcomes.	✓ TEVAR with Gore C-TAG (2012); chimney stent LSA (2012); left carotid–subclavian bypass (2013) — all described with outcomes.	2
**Clinical Findings**				
5	Clinical findings	Describe relevant physical examination (PE) findings.	✓ Hypotension, haemodynamic compromise on presentation. Neurological: acute-onset left facial paresis (transient; brain CT negative for infarction).	2
**Timeline**				
6	Timeline	Depict important dates and times in this case (table or figure is strongly recommended).	✓ Timeline described in narrative: 2012 (TEVAR + chimney stent), 2013 (carotid–subclavian bypass), 2024 (acute re-presentation and emergency surgery), POD 12 (discharge), 6 weeks (early follow-up). A formal timeline table may be added as a figure if requested by the editors.	2
**Diagnostic Assessment**				
7a	Diagnostic assessment	Diagnostic methods (PE, laboratory testing, imaging, surveys).	✓ Contrast-enhanced CTA: Stanford Type A dissection, ascending aorta origin with	2

			extension into prior TEVAR. Cerebral CT: no acute infarction. Preoperative EuroSCORE II: 79.3%.	
**7b**	Diagnostic assessment	Diagnostic challenges (advanced or rare disease, limited resources).	✓ Discussed in Case Presentation and Discussion: rigid endograft complicated standard diagnostic assessment; distinguishing retrograde dissection from primary Type A pathology required high-resolution CTA.	2–3
**7c**	Diagnostic assessment	Diagnosis (including other diagnoses considered).	✓ Stanford Type A aortic dissection (secondary, retrograde) arising 12 years post-TEVAR. Differential diagnosis of acute coronary syndrome excluded by CTA.	2
**7d**	Diagnostic assessment	Prognostic characteristics (staging in oncology) where applicable.	N/A — surgical pathology, not oncology.	—
**Therapeutic Intervention**				
**8a**	Therapeutic intervention	Types of therapeutic intervention (pharmacological, surgical, preventive, self-care).	✓ Emergency surgical intervention: ascending aortic replacement (Dacron graft), AMDS 55/55 (211 mm) deployment, aortic valve resuspension, left atrial appendage resection. Cannulation: right axillary (arterial), right atrium (venous). Bilateral antegrade cerebral perfusion (BCA + LCCA, 13–16 mL/kg/min, 70 mmHg).	2–3
**8b**	Therapeutic intervention	Administration of therapeutic intervention (dosage, strength, duration).	✓ CPB: 139 minutes. Circulatory arrest: 22°C (deep hypothermia), 24 minutes. Device: Gore C-TAG (proximal 37×150 mm, distal 34×150 mm, 2012); AMDS 55/55 × 211 mm (2024). AMDS–TEVAR overlap: 116–131 mm.	2–3
**8c**	Therapeutic intervention	Changes in the therapeutic intervention (with rationale).	✓ The decision to use AMDS instead of FET is discussed with specific rationale: advanced age, high perioperative risk (EuroSCORE II 79.3%), pre-existing endograft precluding standard FET sizing, and absence of SAV dissection.	3–4
**Follow-up and Outcomes**				
**9a**	Follow-up & outcomes	Clinician- and patient-assessed outcomes.	✓ Postoperative: preserved neurological function, haemodynamic stability, no end-organ dysfunction. Follow-up CTA: correct AMDS positioning, false lumen exclusion, stable endograft. Discharged POD 12.	3
**9b**	Follow-up & outcomes	Important follow-up diagnostic and other test results.	✓ Postoperative CTA confirmed patent true lumen perfusion to celiac axis, SMA, bilateral renal arteries. False lumen excluded at visceral level. AMDS–TEVAR integration confirmed.	3
**9c**	Follow-up & outcomes	Intervention adherence and tolerability (how was this assessed)?	✓ Patient tolerated the procedure without intraoperative complications. Uneventful postoperative course. Clinically well at 6-week follow-up.	3
**Patient Perspective**				
**10**	Patient perspective	Did the patient share his or her perspective or experience? (Direct quotes preferred.)	✓ Section 6 (Patient Perspective): patient expressed gratitude for prompt diagnosis and clear communication; reported improved well-being and confidence in the outcome.	5
**Informed Consent**				

**11**	Informed consent	Did the patient give informed consent? Please provide if requested.	✓ Section 7: Written informed consent obtained from the patient for publication. Copy available for editorial review upon request.	5

Reference: Gagnier JJ, Kienle G, Altman DG, Moher D, Sox H, Riley D; CARE Group. The CARE guidelines: consensus-based clinical case reporting guideline development. J Med Case Rep. 2013;7:223. doi: 10.1186/1752-1947-7-223

Corresponding Author: Dr. Gjoko Boshkoski, g.boshkoski@asklepios.com

Institution: Department of Cardiovascular Surgery, Asklepios Clinic Harburg, Hamburg, Germany
